# Life expectancy loss among Native Americans during the COVID-19 pandemic

**DOI:** 10.4054/demres.2022.47.9

**Published:** 2022-07-27

**Authors:** Noreen Goldman, Theresa Andrasfay

**Affiliations:** 1 Office of Population Research and Princeton School of Public and International Affairs, Princeton University, Princeton, NJ, USA.; 2 University of Southern California, Los Angeles, CA, USA

## Abstract

**BACKGROUND:**

There has been little systematic research on the mortality impact of COVID-19 in the Native American population.

**OBJECTIVE:**

We provide estimates of loss of life expectancy in 2020 and 2021 relative to 2019 for the Native American population.

**METHODS:**

We use data on age-specific all-cause mortality rates from CDC WONDER and the 2019 life table recently released by the National Vital Statistics System for Native Americans to calculate life tables for the Native American population in 2020 and 2021 and to obtain estimates of life expectancy reductions during the COVID-19 pandemic.

**RESULTS:**

The pandemic has set Native Americans further behind other major racial/ethnic groups in terms of life expectancy. The estimated loss in life expectancy at birth for Native Americans is 4.5 years in 2020 and 6.4 years in 2021 relative to 2019.

**CONCLUSIONS:**

These results underscore the disproportionate share of deaths experienced by Native Americans: a loss in life expectancy at birth in 2020 that is more than three years above that for Whites and about 1.5 years above the losses for the Black and Latino populations. Despite a successful vaccination campaign among Native Americans, the estimated loss in life expectancy at birth in 2021 unexpectedly exceeds that in 2020.

**CONTRIBUTION:**

The increased loss in life expectancy in 2021, despite higher vaccination rates than in other racial/ethnic groups, highlights the huge challenges faced by Native Americans in their efforts to control the deleterious consequences of the pandemic.

## 1. Introduction

Despite heightened media attention to the large number of Native Americans dying from COVID-19, there has been little systematic research on the mortality impact of the coronavirus in this population. One exception is a recent paper demonstrating that age-standardized COVID-19 mortality rates among Native Americans exceeded corresponding rates for the White, Black, and Latino populations during 2020 (Leggat-Barr, Uchikoshi, and Goldman 2021). A separate study based on similar data, in this case for six racial and ethnic groups, indicated that the age-standardized COVID-19 death rate in 2020 among Native Americans was surpassed only by that for Hawaiian and other Pacific Islanders (Feldman and Bassett 2021).

In contrast to the standardized death rate used in these previous papers, measures of reductions in period life expectancy during the COVID-19 pandemic quantify the impact of the disease in a way that is easy to interpret while still adjusting for a population’s age distribution. Life expectancy at birth denotes the number of years newborns could expect to live if they experienced the age-specific death rates of a given period throughout their lives; similarly, life expectancy at age 65 reflects the expected number of years of life remaining for a person aged 65, again based on death rates for that period. The impact of COVID-19 on mortality of a population, be it a nation or a subgroup, can meaningfully be assessed in terms of declines in life expectancy between a period prior to and one during the pandemic.

Although the impact of COVID-19 on survival for the White, Black, and Latino populations has been assessed in terms of declines in life expectancy (Andrasfay and Goldman 2021a, 2021b; Arias et al. 2021a; Woolf, Masters, and Aron 2021), this useful measure of longevity has not yet been used for Native Americans. The major obstacle has been the paucity of high-quality data. Measures of life expectancy are derived from life tables, which were not available for the total Native American population before those published by the National Vital Statistics System (NVSS) in November 2021 (Arias et al. 2021b). Based on these recent pre-pandemic life tables and published classification ratios to correct for misreporting of American Indian/Alaskan Native (AI/AN) race, we are now able to calculate life tables for the pandemic period.

In this paper, we provide estimates of the loss of life expectancy in 2020 and 2021 for Native American men and women. Native Americans, who constitute about 2% of the US population, are defined as those self-identifying in the US Census as American Indian or Alaskan Native, excluding those also identifying as Latino. The analysis by Leggat-Barr and colleagues (2021) suggests that the mortality impact in 2020 was exceptionally large due to the many risk factors for viral exposure and viral severity present in this population: high poverty rates, crowded living arrangements, low access to quality health care driven in part by low rates of health insurance (aside from that provided by the poorly funded Indian Health Service [IHS]), frequent employment in frontline jobs, and a high prevalence of comorbidities that increase the risk of COVID-19 fatality. The estimated age-standardized COVID-19 death rate for AI/AN in 2020 exceeded that for other racial/ethnic groups nationally and in most of the states analyzed in the study. In addition, despite the implementation of mitigation strategies by Native American communities, such as contact tracing, sealing borders, mask mandates, and enforcement of lockdowns (Foxworth et al. 2021), state-level COVID-19 death rates in 2020 were strongly correlated with the proportion of Native Americans residing on reservations within the state (Leggat-Barr, Uchikoshi, and Goldman 2021).

To the best of our knowledge, no estimates of the overall mortality impact for Native Americans have been published for 2021. Despite the high burden of COVID-19 on the AI/AN population in 2020, there were reasons for optimism for 2021. From the start of vaccine availability, the AI/AN population has had higher rates of vaccine uptake than other racial and ethnic groups (Foxworth et al. 2021). Vaccination efforts that were culturally sensitive, combined with a steady supply of vaccine doses, were instrumental to this achievement. Most notable among these strategies was the use of vaccinated community elders as role models who emphasized the importance of preserving AI/AN culture and protecting Native American communities (Foxworth et al. 2021; Silberner 2021).

To assess the impact of COVID-19 on life expectancy in 2020 and 2021, we use the newly published 2019 life tables for the Native American population to provide information on death rates and life expectancy just prior to the pandemic (Arias et al. 2021b). These life tables have been adjusted by NVSS via linkages between the 2010 census and death registration data to correct for misclassification of race and ethnicity on death certificates, a well-recognized and serious source of bias in data for the Native American population that has resulted in underestimation of mortality (Arias et al. 2021b; Espey et al. 2014; Jim et al. 2014). Incorporating these NVSS adjustments, we use age-specific all-cause mortality rates from 2020 and 2021 for Native Americans to estimate the loss in life expectancy at birth and at age 65 between 2019 and 2020 and between 2019 and 2021 for the Native American population as a whole and separately by sex. These estimates are then compared with published provisional life expectancy estimates for the White, Black, and Latino populations in 2020.

## 2. Data and methods

Counts of deaths from all causes and estimated population sizes by age, sex, race, and ethnicity in 2020 and 2021 were obtained from the CDC WONDER (Wide-ranging Online Data for Epidemiologic Research) database as of May 25, 2022 (CDC WONDER 2022). Deaths for 2020 are considered final, while deaths for 2021 are considered provisional and therefore are subject to reporting and processing delays.

We first adjust the all-cause counts of deaths in 2020 and 2021 to account for underreporting of Native American race on death certificates. We apply the age group and sex-specific classification ratios published by NVSS (Arias et al. 2021b) to these death counts, with the assumption that the degree and age pattern of misclassification in 2020 and 2021 are equivalent to those in ratios used by NVSS in 2019. The average NVSS classification ratio was 1.34, with slightly higher values for males than females and with the largest values at ages 45–74. We also borrow from the NVSS 2019 life tables the _1_a_0_ values, which are the average numbers of person-years lived by infants who died before their first birthday. We use the adjusted death counts to estimate age-specific mortality rates (_n_M_x_) for both years and, subsequently, estimate the remaining life table columns using standard life table relationships (Preston, Heuveline, and Guillot 2000). For comparison, we present published life expectancy values for 2019 (Arias and Xu 2022) and provisional estimates for 2020 (Arias et al. 2021a) for the total US population and the Latino, White, and Black populations by sex.

## 3. Results

Simple descriptive statistics of COVID-19 deaths among Native Americans provide insights into changes in life expectancy during the pandemic. Before adjustment for racial misclassification, there were 4,265 Native American deaths in 2020 and 4,615 in 2021 for which COVID-19 was the underlying cause. The median ages of these deaths declined from 69 to 65 years over this period, which, combined with the increase in numbers of deaths, suggests, contrary to expectation, that the loss in life expectancy at birth may be greater in 2021.

**Figure 1:**
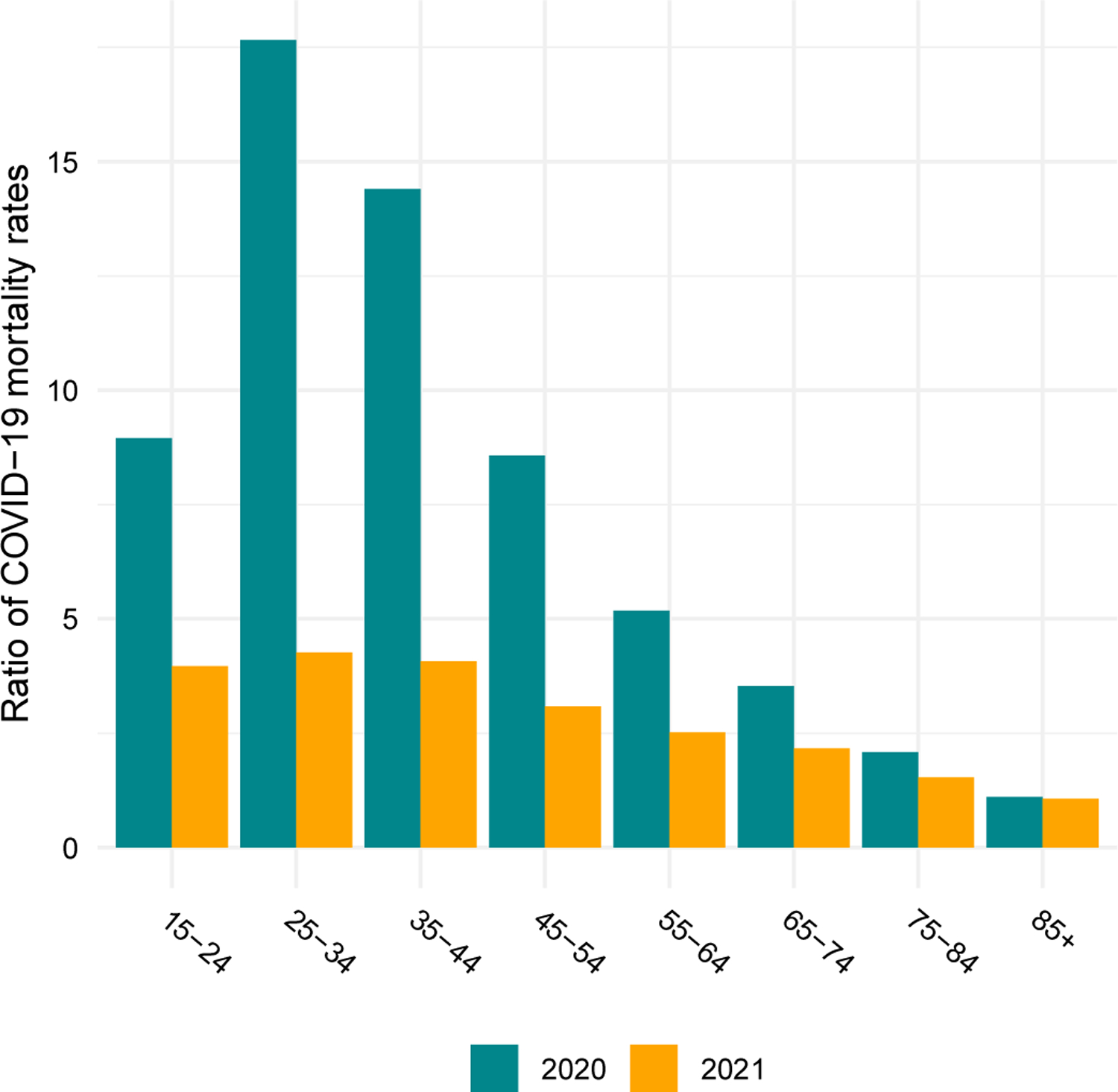
Ratio of Native American to White COVID-19 age-specific mortality rates in 2020 and 2021 *Note*: Data are from CDC WONDER and include all deaths for which COVID-19 is the underlying cause of death. Native American COVID-19 death counts have been adjusted to account for misreporting of AI/AN race on death certificates. Ratios below age 15 are not shown because counts of COVID-19 deaths are suppressed at these ages for confidentiality.

Due partly to the younger age distribution of the Native American population compared to the White population (Akee and Reber 2021; Leggat-Barr, Uchikoshi, and Goldman 2021), the median ages of COVID-19 deaths are more than a decade higher for Whites – 82 in 2020 and 75 in 2021 – than for Native Americans. However, as shown in Figure 1, these differences in ages at death primarily reflect the much higher COVID-19 mortality rates among Native Americans at younger ages. COVID-19 death rates in the young adult and middle age range are about ten or more times as high for Native Americans than for Whites in 2020 and are reduced to about four to five times as high in 2021. Above age 75, death rates are approximately twice as high among Native Americans in both years. Preliminary work suggests that the Black and Latino populations also experienced reductions in age-specific death rates relative to Whites from 2020 to 2021, driven partly by rising mortality rates among Whites (Andrasfay and Goldman 2022).

Table 1 presents life expectancy at birth (e_0_) and at age 65 (e_65_) by sex as calculated by NVSS in its 2019 life tables, along with our results for 2020 and 2021. The estimated declines in life expectancy at birth are 4.5 years in 2020 and a substantially larger 6.4 years in 2021. The pandemic reduced Native American life expectancy at birth from the already low value of 72 years in 2019 to about 67 years in 2020 and about 65 years in 2021. The larger reductions for men than for women have resulted in a gender gap in life expectancy at birth estimated at 7.2 years in 2021 compared with 6.4 years in 2019 and are consistent with larger reductions seen among men in other populations in the United States. The estimated impact of the pandemic on e_65_ is about two years in both 2020 and 2021, slightly higher for women than men, in contrast to the gender difference in loss of life expectancy at birth.

**Figure 2:**
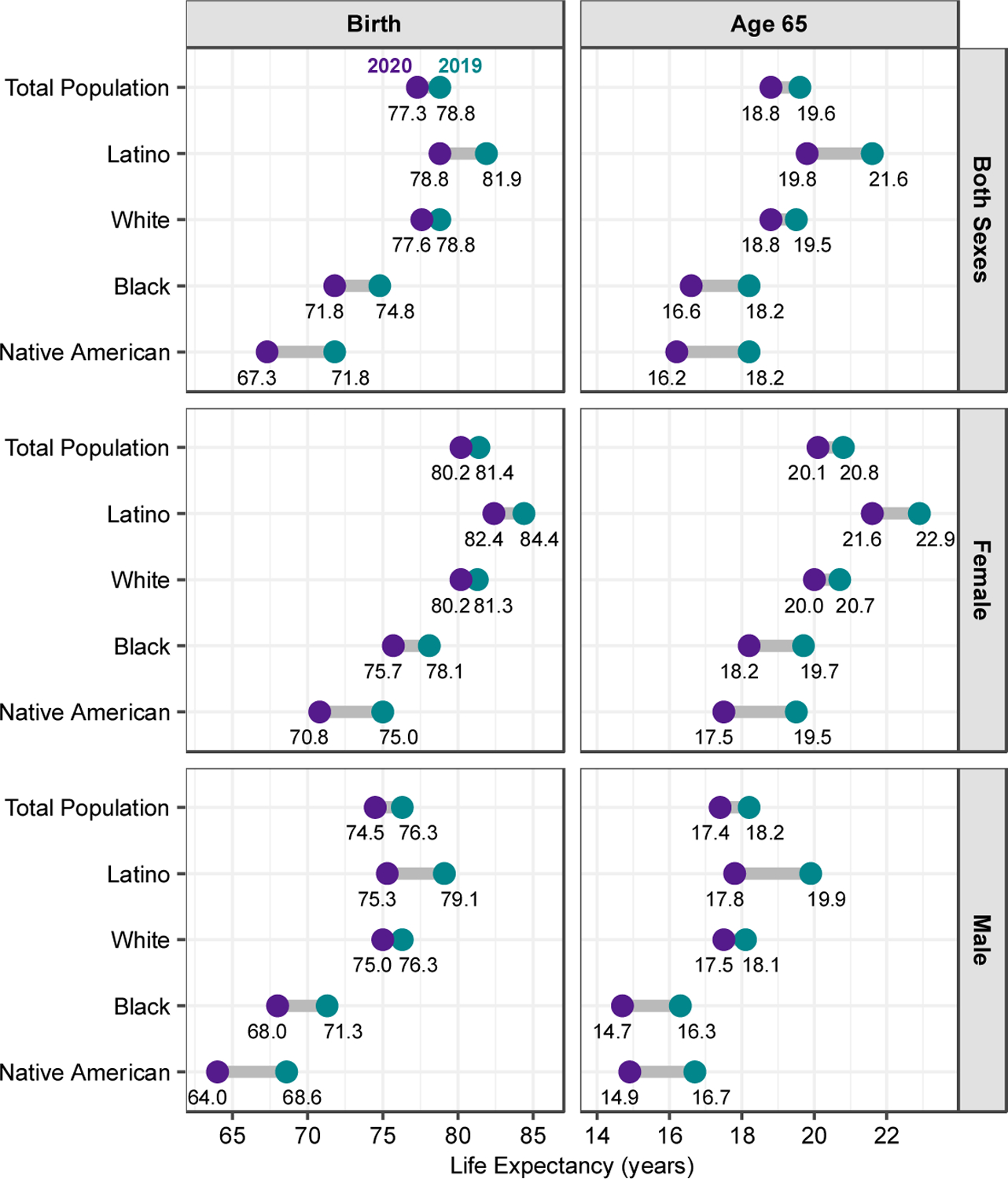
Life expectancy at birth and at age 65 by race/ethnicity, estimates for 2020 compared to 2019 *Note*: Life expectancy values for the total, Latino, White, and Black populations are taken from published NVSS provisional estimates. Estimated 2020 life expectancy values for the Native American population are the authors’ calculations from CDC WONDER mortality data and incorporate adjustments for racial misclassification of deaths of Native Americans.

Figure 2 compares the estimated loss in life expectancy by sex between 2019 and 2020 across racial and ethnic groups, based on our estimates for Native Americans and those from Arias et al. (2021a) for the Latino, White, and Black populations. This figure shows that, even before the pandemic, the Native American population had much lower life expectancy than other major racial/ethnic groups in the United States. The pre-pandemic (2019) e_0_ of 71.8 for the AI/AN population was seven years below that of the White population and a decade lower than that of the Latino population. The pandemic has set the AI/AN population behind even further: The estimated loss in e_0_ for Native American men and women in 2020 greatly exceeds that for all the other groups. For both sexes combined, the loss in e_0_ for Native Americans of 4.5 years is more than three years above that for Whites and about 1.5 years above those for the Black and Latino populations. The two-year loss in e_65_ for Native Americans (both sexes combined) also exceeds the corresponding declines for the other racial/ethnic groups. Only Latino men experienced a greater loss in e_65_ than Native American men.

## 4. Discussion

Throughout the pandemic, scholars and journalists have acknowledged the disproportionate share of COVID-19 deaths experienced by Native Americans. Here we quantify this mortality burden in terms of reductions in life expectancy at birth and at age 65 in a way that takes into account both deaths from COVID-19 and excess deaths from other causes. We estimate that life expectancy at birth in 2020 and 2021 has declined to approximately 67 and 65 years, respectively, shockingly low values in a high-income country. These levels of life expectancy are far below those in every country in the Americas with the sole exception of Haiti (where the estimated life expectancy is similar at 64) and are several years below values observed prior to the pandemic in India, Pakistan, and Nepal (Population Reference Bureau 2020). Mortality during the pandemic has exacerbated an already stark mortality disparity between Whites and Native Americans in the United States, increasing a seven-year gap in life expectancy prior to the pandemic to ten years in 2020. Our estimates suggest that reductions in 2020 life expectancy at birth were almost four times as large for Native Americans as they were for Whites. Although much of this decline resulted directly from COVID-19 deaths, mortality from non-COVID-19 causes also increased substantially during the pandemic: Death rates from all non-COVID-19 causes combined rose progressively through the three years for all adult age groups, with the largest proportional increases between ages 25 and 44 (authors’ calculations from CDC WONDER 2022).

One limitation of this analysis is that our estimates for 2021 are based on provisional counts of death, as are the comparisons for other racial/ethnic groups in 2020. A second limitation is that our estimates of life expectancy for 2020 and 2021 depend heavily on NVSS classification ratios derived from the matching of census and death certificate information a decade earlier (2010–2011). The adjustment factor (1.34 on average) results primarily from underreporting of Native American race on death certificates, with most misclassified Native American decedents reported as White on death certificates (Arias et al. 2021b). Such misclassification has been especially prevalent for persons self-identifying with multiple races in census data, particularly among AI/AN people residing outside IHS contract delivery areas (Jim et al. 2014). To the extent that misclassification of race/ethnicity has changed over the past decade, these estimates of life expectancy will be biased (for example, too low if underreporting of Native American race on death certificates has diminished). However, because we use the same classification ratios in 2019, 2020, and 2021, our estimates of *loss* in life expectancy over this period are likely robust to the extent that these ratios remained constant over the three years.

The increase in mortality rates during the pandemic among Native Americans was likely due in large part to the high rates of chronic disease in this population prior to the pandemic. Not only did the high prevalence of comorbidities in the AI/AN population increase susceptibility to COVID-19 infection and severity, but COVID-19 infection and potential experiences with ‘long COVID’ likely increased the severity and hence fatality of non-COVID-19 illnesses due to the physiological sequelae of the infection and reduced availability and use of health care during the pandemic (Leggat-Barr, Uchikoshi, and Goldman 2021). As with the US population at large, it is also likely that detrimental health-related behaviors, such as smoking, drinking, and drug use, became more frequent during this time (Kalkhoran, Levy, and Rigotti 2021; Zhang et al. 2021). There is already evidence of a more than 40% increase in fatal drug overdoses among Native Americans in 2020, a number that exceeds increases for Whites and Latinos (Friedman and Hansen 2022; Hedegaard et al. 2021).

Despite the successful vaccination campaign among Native Americans, as well as efforts to increase COVID-19 testing and to modernize the health IT infrastructure of the IHS (Indian Health Service 2022), the estimated loss in life expectancy at birth in 2021 from pre-pandemic levels substantially exceeds that in 2020. There are several plausible explanations for this unexpected and disturbing finding. First, because the initial vaccine rollout prioritized health care workers, no racial/ethnic group had substantial vaccination coverage during January–February 2021 – two of the deadliest months of the pandemic (Centers for Disease Control and Prevention 2022; Ndugga et al. 2021). Second, two highly contagious variants that partially evaded vaccine-acquired and natural immunity, Delta and Omicron, emerged in 2021 (Kupferschmidt and Wadman 2021; del Rio, Omer, and Malani 2021). Third, the Native American population, like the general US population, still has a substantial proportion of unvaccinated persons, especially among younger adults, and a relatively low uptake of booster doses (Centers for Disease Control and Prevention 2022). Fourth, death rates from several chronic conditions and drug overdoses increased during the pandemic, and these increases did not abate in 2021. Fifth, and most importantly, Native Americans continue to experience large social, economic, and health inequities, some of which have persisted for centuries. These factors increase risks of COVID-19 infection, hospitalization, and death: high rates of poverty; poor housing infrastructure, including inadequate plumbing; crowded living conditions, often involving multigenerational families; employment in low-income frontline jobs that cannot be performed remotely; high rates of comorbidities (particularly obesity and diabetes) that increase the severity and fatality of COVID-19; a high prevalence of smoking; low rates of health insurance other than that provided by IHS; and poorly resourced health care systems that provide inadequate and often inaccessible care (Leggat-Barr, Uchikoshi, and Goldman 2021). Because many of these risk factors are more prevalent in tribal lands than in nontribal areas, vulnerability to COVID-19 is especially high for Native Americans residing on reservations (Leggat-Barr, Uchikoshi, and Goldman 2021).

Although COVID-19 death rates among Native Americans undoubtedly would have been larger in the absence of a successful vaccine campaign, our results underscore the huge challenges faced by this population in its efforts to control the appalling consequences of the ongoing pandemic, which coexist with high rates of morbidity and mortality from other causes. The large financial investment in the American Rescue Plan to enhance identification and treatment of COVID-19 infections and to strengthen the public health infrastructure for the Native American population is a significant step forward (Indian Health Service 2021).

## Supplementary Material

Supplementary File

## Figures and Tables

**Table 1:** Estimates of life expectancy and losses in life expectancy for the Native American population

	Birth (e_0_)			Age 65 (e_65_)		
	Both Sexes	Female	Male	Both Sexes	Female	Male
**2019**						
Life expectancy	71.8	75.0	68.6	18.2	19.5	16.7
**2020**						
COVID-19 deaths			4,265			
Life expectancy	67.3	70.8	64.0	16.2	17.5	14.9
Reduction in ex from 2019	4.5	4.2	4.6	2.0	2.0	1.8
**2021**						
COVID-19 deaths			4,615			
Life expectancy	65.4	69.2	62.0	16.1	17.1	14.9
Reduction in ex from 2019	6.4	5.8	6.6	2.1	2.4	1.8

*Notes*: Apart from life expectancy values from 2019 provided by NVSS, all life expectancy estimates are the authors’ calculations. Estimates for 2020 and 2021 are based on all-cause mortality data provided by CDC WONDER and are considered provisional for 2021. Life expectancy estimates for Native Americans have been adjusted for racial misclassification using classification ratios published by NVSS (2021).
